# Whole Body Phase Angle as a Promising Marker of Disease Severity in Facioscapulohumeral Muscular Dystrophy

**DOI:** 10.1002/mus.70391

**Published:** 2026-08-31

**Authors:** Oscar Crisafulli, Renato Baptista, Andi Nuredini, Matteo Fortunati, Luca Grattarola, Rossella Tupler, Anna Odone, Giuseppe D'Antona

**Affiliations:** ^1^ CRIAMS‐Sport Medicine Centre Voghera, University of Pavia Voghera Italy; ^2^ Department of Research and Development LUNEX Differdange Luxembourg; ^3^ Luxembourg Health & Sport Sciences Research Institute A.S.B.l. Differdange Luxembourg; ^4^ Department of Biomedical, Metabolic and Neural Sciences University of Modena and Reggio Emilia Modena Italy; ^5^ Department of Industrial Engineering University of Tor Vergata Rome Italy; ^6^ Department of Public Health, Experimental and Forensic Medicine University of Pavia Pavia Italy; ^7^ Medical Direction, Fondazione IRCCS Policlinico San Matteo Pavia Italy

**Keywords:** bioelectrical impedance analysis, body composition, clinical score, disease severity, facioscapulohumeral muscular dystrophy, phase angle

## Abstract

**Introduction/Aims:**

To investigate its potential role as a marker of disease severity in facioscapulohumeral dystrophy (FSHD), this study examined the association between whole‐body phase angle (PhA) and clinically assessed severity in FSHD patients.

**Methods:**

Patients were evaluated for PhA using bioelectrical impedance analysis (BIA) and for disease severity using the FSHD clinical score. In a subgroup, physical activity level (PAL) was assessed using a questionnaire. Pearson correlations were performed in the total cohort and PAL‐assessed subgroup, stratified by sex and clinical phenotype (category A and non‐A) according to the Comprehensive Clinical Evaluation Form. Multivariate regression analyses were conducted adjusting for age, body mass index, and PAL. Due to the small sample, correlation was not performed in PAL‐assessed non‐A patients, while multivariate regression was not performed in non‐A patients and PAL‐assessed stratified subgroups.

**Results:**

Seventy‐two patients (mean age 39.94 ± 17.52 years; 27 females and 45 males; 58 category A and 14 non‐A) were enrolled. The PAL‐assessed subgroup comprised 38 patients (mean age 41.39 ± 14.98 years; 16 females and 22 males; 31 category A and 7 non‐A). Pearson analyses revealed that PhA was inversely associated with clinical score in all groups (*r* –0.63 to –0.84, *p* 0.02 to < 0.001). Multivariate regression confirmed PhA as the sole independent predictor across all groups (*β* = –0.64 to –0.93, all *p* < 0.001).

**Discussion:**

These results support the potential role of PhA as a marker of disease severity in FSHD. Longitudinal studies are warranted to determine its sensitivity for monitoring disease progression over time.

## 1 Introduction

Facioscapulohumeral muscular dystrophy (FSHD) causes progressive muscle loss accompanied by an increase in adipose tissue [1]. These body composition changes lead to muscle weakness and functional limitations [2]. Functional‐based clinical scales have been developed to provide numerical measures of disease severity [3, 4]. However, these assessments may be influenced by patient cooperation and learning effects [5]. Moreover, given the slow disease progression, clinical scales may lack sensitivity to detect subtle changes over time and reliably capture treatment effects in clinical trials [5]. Parameters obtained from body composition assessments, such as magnetic resonance imaging (MRI), ultrasound, and dual‐energy X‐ray absorptiometry (DXA), have shown good correlation with clinical assessment scales, supporting their role in disease evaluation [5]. However, routine clinical use and follow‐up can be limited by cost and access (MRI), inter‐device variability (DXA), and operator dependency (ultrasound) [6]. The potential utility of bioelectrical impedance analysis (BIA)‐derived Phase Angle (PhA), a raw parameter reflecting whole body tissues' resistance and reactance [7], remains unexplored in FSHD. Although PhA has been associated with functional measures [8, 9] and muscle quality [10] in sarcopenic cohorts, and with disease severity in myotonic dystrophy Type 1 [11], in FSHD it is only known to distinguish between patients and healthy controls [12, 13], while its association with clinical scales has not been investigated. The identification of an association between PhA and clinical assessment scores could provide a novel, accessible, and cost‐effective marker of disease severity with potential applicability in routine practice. To address this, the present report analyzes the association between PhA and disease severity in a cohort of FSHD patients, accounting for potential confounding factors that may influence this relationship.

## 2 Methods

### 2.1 Participants and Procedures

The study was conducted at the CRIAMS–Sport Medicine Centre, University of Pavia (Voghera, Italy), approved by the Lombardy Territorial Ethics Committee 6 (protocol no. 0006176/24), and performed in accordance with the Declaration of Helsinki. Written informed consent was obtained from all participants or, in the case of minors, from their parents or legal guardians. Patients, identified and recruited among those regularly followed at our center, were enrolled if they had a clinical and genetic diagnosis of FSHD and were registered in the Italian National FSHD Registry [14]. Exclusion criteria included comorbid cardiovascular, pulmonary, orthopedic, or neuromuscular disorders other than FSHD. Patients were classified according to the Comprehensive Clinical Evaluation Form (CCEF) into categories A (typical phenotype), B (incomplete phenotype), C (asymptomatic), or D (complex/uncommon phenotype) [15]. In a single session, patients underwent assessments of disease severity and PhA. In a subgroup, physical activity level (PAL) was also evaluated.

Disease severity was quantified using the FSHD clinical score [4], corresponding to Section 2 of the CCEF [15], ranging from 0 (no motor impairment) to 15 (severe functional impairment). The assessment was performed by a neurologist with extensive experience in the clinical evaluation of FSHD patients (AN). PhA was assessed by BIA as previously described [16]. Briefly, a single‐frequency (50 kHz) impedance analyzer was used (BIA 101 BIVA PRO, Akern, Florence, Italy) under standardized conditions. Two adhesive electrodes (Biatrodes, Akern Srl, Florence, Italy) were applied to the dorsum of the hand and foot on the same body side. A low‐amplitude alternating current was passed through the body, providing whole‐body measurements of resistance (*R*) and reactance (*Xc*), from which PhA (°) was calculated as arctangent (*Xc*/*R*) × 180°/*π*. Finally, PAL was assessed using the International Physical Activity Questionnaire (IPAQ) and expressed as metabolic equivalents of task minutes per week (METs‐min/week), a validated approach previously applied in FSHD [16, 17].

### 2.2 Statistical Analyses

Statistical analyses were conducted using SPSS version 31.0 (IBM, Armonk, NY). Associations between PhA and clinical score were initially examined using Pearson's correlation coefficients, with 95% confidence intervals (CI) calculated via Fisher's r‐to‐z transformation and back‐transformed to the r scale. Analyses were performed in the full sample and stratified by sex as a potential confounder, given previously reported sex‐related differences in PhA values [18]. A secondary analysis stratified participants into category A (typical clinical phenotype) and non‐A category (B and D categories; atypical clinical phenotypes combined due to limited sample sizes). This exploratory stratification was based on the hypothesis that distinct FSHD phenotypes may exhibit different patterns of muscle involvement, potentially affecting both disease severity and body composition, and influencing the PhA–clinical score association. The same analytical approach was applied to the subgroup with PAL assessment, except for non‐A patients, who were excluded due to limited sample size. To assess the independent association between PhA and clinical score, multivariable linear regression analyses were conducted with clinical score as the dependent variable. The number of covariates included in each regression model was determined a priori based on sample size considerations, using a conservative target of approximately 10 observations per predictor variable to reduce the risk of overfitting, ensuring stability and interpretability of the estimates [19]. Model fit was evaluated using *R* ^2^ and adjusted *R* ^2^. Regression results are reported as unstandardized coefficients (B), standardized coefficients (*β*), *p*‐values, and 95% CI. Collinearity diagnostics indicated acceptable multicollinearity (maximum variance inflation factor = 2.1), supporting the stability and interpretability of the estimates [20]. The primary model included all participants and was adjusted for potential confounders, including age [21] and body mass index (BMI) [22]. Additional analyses were conducted stratifying by sex and restricting the sample to patients in category A, as the number of non‐A participants was insufficient to support reliable modeling. In a subsample of participants, a secondary regression model incorporated PAL as an additional covariate [23]. These analyses were performed only in the overall PAL subgroup as sex and phenotype stratification were not feasible due to limited sample size. Statistical significance was defined as *α* = 0.05 (two‐tailed).

## 3 Results

A total of 72 FSHD Type 1 patients participated in the study. Details on sample size, sex distribution, age, and anthropometric characteristics for the overall sample and subgroups are summarized in Table 1. The same data for the PAL‐assessed subgroup are available in Table S1.

**TABLE 1:** Descriptive statistics of the overall cohort as a whole group and stratified by sex and clinical phenotype.

	Whole group	Stratification by sex		Stratification by phenotype	
	Total of subjects (*N* = 72)	Male (*N* = 45)	Female (*N* = 27)	Category A (*N* = 58)	Non‐A category (*N* = 14; of which 12 B and 2 D)
Sex					
Female	27 (38%)	—	27 (100%)	26 (45%)	2 (16%)
Male	45 (62%)	45 (100%)	—	32 (55%)	12 (84%)
Age (years)					
Mean (SD)	39.94 (17.52)	39.08 (17.79)	41.37 (17.30)	37.91 (17.78)	48.35 (14.01)
Anthropometric characteristics					
Weight (kg)					
Mean (SD)	69.65 (15.69)	73.83 (16.32)	62.72 (11.90)	68.05 (15.53)	76.35 (15.07)
Height (m)					
Mean (SD)	1.70 (0.09)	1.73 (0.09)	1.65 (0.06)	1.69 (0.09)	1.74 (0.10)
Body mass index (kg/m^2^)					
Mean (SD)	23.67 (4.08)	24.18 (4.23)	22.81 (3.73)	23.37 (4.12)	24.91 (3.79)
Phase angle (°)					
Mean (SD)	5.42 (1.21)	5.61 (1.29)	5.10 (0.99)	5.24 (1.19)	6.18 (0.98)
Clinical score (pts)					
Mean (SD)	5.11 (2.81)	4.91 (2.82)	5.44 (2.81)	5.72 (2.73)	2.57 (1.39)

*Note:* Data are expressed as frequencies and mean (standard deviation).

Abbreviations: °, degree; B, category B of the FSHD Comprehensive Clinical Evaluation Form; BMI, body mass index; D, category D of the FSHD Comprehensive Clinical Evaluation Form; kg, kilograms; m, meters; pts, points; SD, standard deviation.

PhA was strongly and inversely associated with clinical score across the whole cohort (*r* = −0.78, *p* < 0.001, 95% CI [−0.86, −0.67]). This association remained significant when stratified by sex and clinical category.

Similarly, in the subgroup with PAL assessment, the association was strong and significant for the whole group (*r* = −0.76, *p* < 0.001, 95% CI [−0.87, −0.57]), stratifying by sex and category A patients. The subgroup‐specific associations are graphically presented in Figure 1.

**FIGURE 1:**
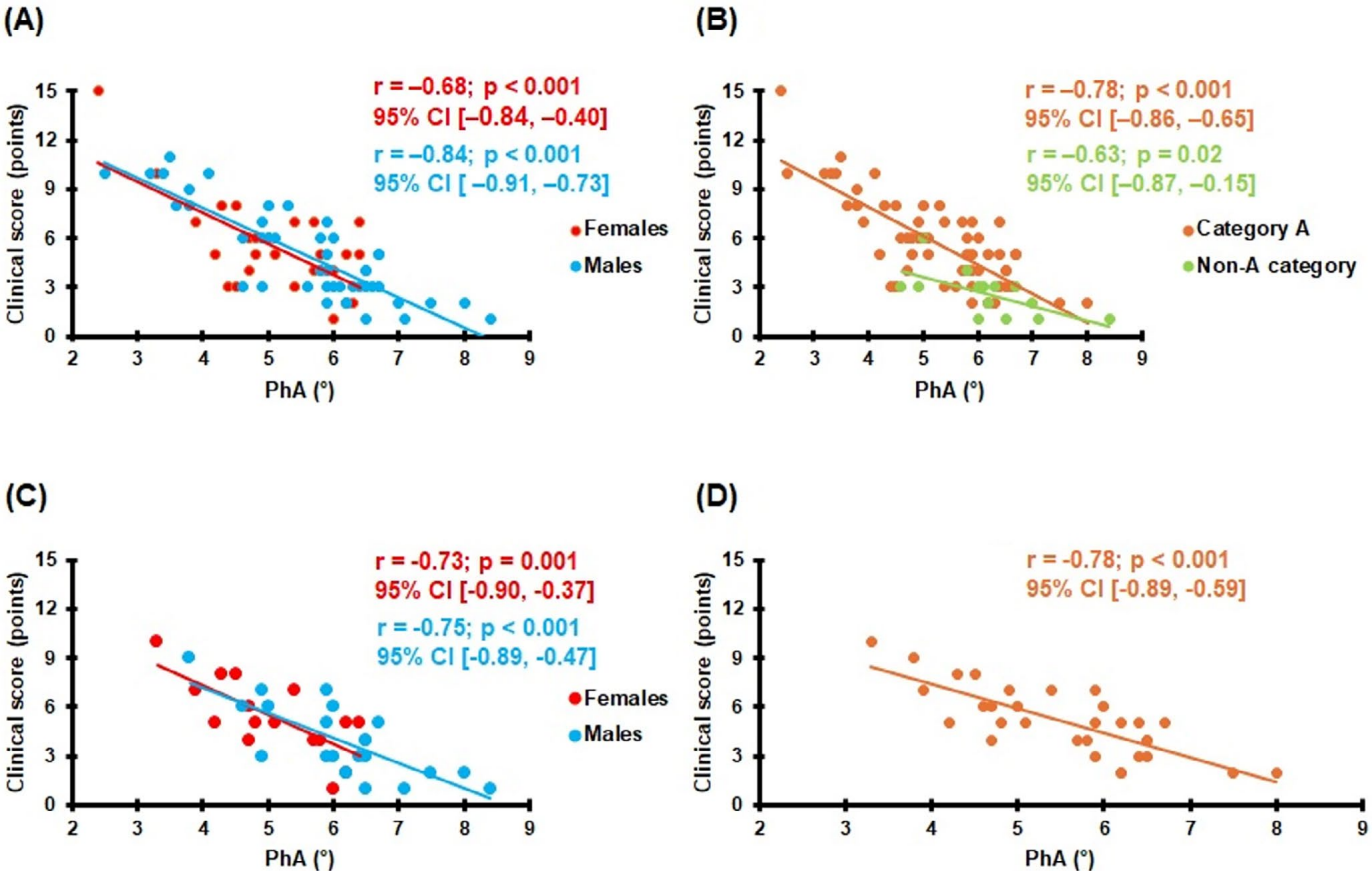
Graphical representation of Pearson's correlation analyses between phase angle (PhA) and clinical score across different groups. Panels A and B show correlations stratified by sex (females *N* = 27, males *N* = 45) and clinical phenotype (category A *N* = 58, non‐A *N* = 14), respectively, of the whole sample. Following the same sequence, while Panels C and D show correlations stratified by sex (females *N* = 16, males *N* = 22) and category A (*N* = 31), respectively, of patients with physical activity level (PAL) assessment. As specified in the text, non‐A patients within the PAL subgroup were not analyzed due to the small sample size (*N* = 7). All correlations are non‐partial and unadjusted for covariates. CI, confidence interval; PAL, physical activity level; PhA, phase angle.

Multivariable regression analyses provided coherent observations. PhA was the only independent predictor of clinical score in the whole sample and in the PAL subgroup.

Sex stratification yielded consistent results, with PhA remaining the only significant predictor of clinical score in both males and females.

The association observed in category A patients was also confirmed. Detailed results of all regression models are summarized in Table 2.

**TABLE 2:** Multivariable associations between clinical score and predictors in the overall cohort and subgroups.

Outcome	Group	*R* ^2^	Adj. *R* ^2^	Phase angle *β* (*B*, *p*, [95% CI])	Age *β* (*B*, *p*, [95% CI])	BMI *β* (*B*, *p*, [95% CI])	Physical activity level *β* (*B*, *p*, [95% CI])
Whole group							
Clinical score	Total of subjects (*N* = 72)	0.63	0.61	−0.78 (*B* = −1.81, ***p* < 0.001**, [−2.19, −1.43])	−0.06 (*B* = −0.01, *p* = 0.51, [−0.04, 0.02])	−0.1 (*B* = −0.07, *p* = 0.25, [−0.19, 0.05])	
Stratification by sex							
—	Male (*N* = 45)	0.72	0.70	−0.88 (*B* = −1.92, ***p* < 0.001**, [−2.33, −1.51])	−0.14 (*B* = −0.02, *p* = 0.23, [−0.06, 0.01])	0.03 (*B* = 0.02, *p* = 0.79, [−0.13, 0.17])	
—	Female (*N* = 27)	0.54	0.48	−0.64 (*B* = −1.81, ***p* < 0.001**, [−2.7, −0.92])	−0.04 (*B* = −0.007, *p* = 0.78, [−0.06, 0.04])	−0.29 (*B* = −0.22, *p* = 0.06, [−0.19, 0.05])	
Stratification by phenotype							
Clinical score	Category A (*N* = 58)	0.62	0.6	−0.74 (*B* = −1.7, ***p* < 0.001**, [−2.13, −1.26])	0.04 (*B* = 0.006, *p* = 0.7, [−0.03, 0.04])	−0.14 (*B* = −0.09, *p* = 0.15, [−0.22, 0.04])	
Subgroup with physical activity level assessment							
Clinical score	Total of subjects (*N* = 38)	0.63	0.59	−0.93 (*B* = −1.91, ***p* < 0.001**, [−2.56, −1.27])	−0.28 (*B* = −0.04, *p* = 0.08, [−0.09, 0.006])	0.09 (*B* = 0.07, *p* = 0.49, [−0.13, 0.27])	−0.06 (*B* = −6.9 × 10^−5^, *p* = 0.62, [0, 0])

*Note:* Statistically significant *p*‐values are highlighted in bold.

Abbreviations: Adj, adjusted; BMI, body mass index; CI, confidence interval; FSHD, facioscapulohumeral dystrophy; *N* number.

## 4 Discussion

Overall, the results indicate that whole‐body PhA is inversely and independently associated with FSHD clinical score. Previous studies using electrical impedance myography (EIM) have demonstrated that localized impedance parameters provide quantitative information on muscle composition and function [24, 25, 26] and are sensitive to disease progression in FSHD [27]. While EIM assesses impedance at the level of individual muscles, whole‐body BIA provides a global assessment of body impedance, from which PhA is derived. Our findings further support the role of impedance analysis in FSHD by showing that whole‐body PhA is significantly associated with clinically assessed disease severity. Whether such parameter is similarly sensitive to disease progression, as previously demonstrated for EIM‐derived measures [27], warrants investigation in longitudinal studies. Given the descriptive and observational nature of this study, a mechanistic explanation cannot be provided. However, it can be hypothesized that progressive muscle wasting and fibro‐fatty replacement [1] may modify the electrical properties of body tissues, contributing to associated alterations in PhA. Nevertheless, our findings seem in line with previous investigations in which PhA was associated with functional capacity in various populations, including sarcopenic older adults [28], stroke patients [29], and hemodialysis patients [30]. Future studies are needed to elucidate the physiological mechanisms underlying this association.

This study has some limitations. Its cross‐sectional design precludes causal inference and limits interpretation as indicative of clinical validity. Multivariable models in females and PAL subgroups (Table 2) should be considered exploratory due to sample size constraints. The association between PhA and disease severity was assessed exclusively using the FSHD clinical score. Future studies should include objective functional assessments, such as muscle strength, to further explore the clinical relevance of PhA. Additionally, PAL was assessed via a self‐reported questionnaire, which may be subject to recall bias.

In conclusion, whole‐body PhA is inversely associated with disease severity, independently of sex, age, BMI, and PAL. These findings support its role as a promising marker of disease severity in FSHD, with potential application in routine clinical monitoring. Longitudinal studies are needed to establish its sensitivity and effectiveness in tracking disease progression over time.

## Author Contributions

O.C. contributed to the conception and design of the study, data acquisition, analysis and interpretation, and drafted the manuscript. R.B. contributed to the statistical analysis. A.N., L.G. and M.F. contributed to data acquisition and interpretation. R.T. and A.O. contributed to data acquisition and interpretation and assisted in writing and revising the manuscript for intellectual content. G.D. contributed to the conception and design of the study, funding acquisition, data analysis and interpretation, drafted the manuscript, and coordinated and supervised the project. All authors critically revised the manuscript, approved the final version, and agreed to be accountable for all aspects of the work, thereby ensuring its integrity and accuracy.

## Funding

This work was supported by the French Muscular Dystrophy Association [Grant n° 24420] to GD.

## Ethics Statement

The study was approved by the Lombardy Territorial Ethics Committee 6 (protocol no. 0006176/24). We confirm that we have read the Journal's position on issues involved in ethical publication and affirm that this report is consistent with those guidelines.

## Consent

Written informed consent was obtained from all participants or, in the case of minors, from their parents or legal guardians.

## Conflicts of Interest

The authors declare no conflicts of interest.

## Supporting information


**Table S1:** Descriptive statistics of the PAL‐assessed cohort as whole group and stratified by sex and clinical phenotype.

## Data Availability

The data that support the findings of this study are available from the corresponding author upon reasonable request.

## References

[1] K. A. Vera , M. McConville , M. Kyba , and M. L. Keller‐Ross , “Sarcopenic Obesity in Facioscapulohumeral Muscular Dystrophy,” Frontiers in Physiology 11 (2020): 1008, 10.3389/fphys.2020.01008. PMC7435048 32903446

[2] A. J. Skalsky , R. T. Abresch , J. J. Han , C. S. Shin , and C. M. McDonald , “The Relationship Between Regional Body Composition and Quantitative Strength in Facioscapulohumeral Muscular Dystrophy (FSHD),” Neuromuscular Disorders 18, no. 11 (2008): 873–880, 10.1016/j.nmd.2008.07.005. 18818077

[3] E. Ricci , G. Galluzzi , G. Deidda , et al., “Progress in the Molecular Diagnosis of Facioscapulohumeral Muscular Dystrophy and Correlation Between the Number of KpnI Repeats at the 4q35 Locus and Clinical Phenotype,” Annals of Neurology 45, no. 6 (1999): 751–757, 10.1002/1531-8249(199906)45:6<751::aid-ana9>3.0.co;2-m. 10360767

[4] C. Lamperti , G. Fabbri , L. Vercelli , et al., “A Standardized Clinical Evaluation of Patients Affected by Facioscapulohumeral Muscular Dystrophy: The FSHD Clinical Score,” Muscle & Nerve 42, no. 2 (2010): 213–217, 10.1002/mus.21671. 20544930

[5] S. C. C. Vincenten , S. Teeselink , K. Mul , et al., “Muscle Imaging in Facioscapulohumeral Muscular Dystrophy Research: A Scoping Review and Expert Recommendations,” Neuromuscular Disorders 47 (2025): 105274, 10.1016/j.nmd.2025.105274. 39884029

[6] F. Ponti , A. De Cinque , N. Fazio , A. Napoli , G. Guglielmi , and A. Bazzocchi , “Ultrasound Imaging, a Stethoscope for Body Composition Assessment,” Quantitative Imaging in Medicine and Surgery 10, no. 8 (2020): 1699–1722, 10.21037/qims-19-1048. PMC7378096 32742962

[7] J. P. D. Costa Pereira , A. S. Rebouças , C. M. Prado , et al., “Phase Angle as a Marker of Muscle Quality: A Systematic Review and Meta‐Analysis,” Clinical Nutrition 43, no. 12 (2024): 308–326, 10.1016/j.clnu.2024.11.008. 39549478

[8] D. D. Espirito Santo Silva , D. L. Waitzberg , R. Passos de Jesus , L. P. M. Oliveira , R. S. Torrinhas , and G. Belarmino , “Phase Angle as a Marker for Sarcopenia in Cirrhosis,” Clinical Nutrition ESPEN 32 (2019): 56–60, 10.1016/j.clnesp.2019.05.003. 31221291

[9] N. M. Santana , C. P. S. Pinho , C. P. da Silva , N. F. Dos Santos , and R. M. L. Mendes , “Phase Angle as a Sarcopenia Marker in Hospitalized Elderly Patients,” Nutrition in Clinical Practice 33, no. 2 (2018): 232–237, 10.1002/ncp.10016. 29397040

[10] O. Di Vincenzo , M. Marra , A. Di Gregorio , F. Pasanisi , and L. Scalfi , “Bioelectrical Impedance Analysis (BIA) ‐Derived Phase Angle in Sarcopenia: A Systematic Review,” Clinical Nutrition 40, no. 5 (2021): 3052–3061, 10.1016/j.clnu.2020.10.048. 33183880

[11] E. Rinninella , G. Silvestri , M. Cintoni , et al., “Clinical Use of Bioelectrical Impedance Analysis in Patients Affected by Myotonic Dystrophy Type 1: A Cross‐Sectional Study,” Nutrition 67–68 (2019): 110546, 10.1016/j.nut.2019.05.012. 31376676

[12] O. Crisafulli , R. Baptista , P. Drid , et al., “Analysis of Body Fluid Distribution, Phase Angle and Its Association With Maximal Oxygen Consumption in Facioscapulohumeral Dystrophy: An Observational Study,” Health Science Reports 8, no. 1 (2025): e70335, 10.1002/hsr2.70335. PMC11726644 39807483

[13] O. Crisafulli , G. Bottoni , J. Lacetera , et al., “Bioimpedance Analysis of Fat Free Mass and Its Subcomponents and Relative Associations With Maximal Oxygen Consumption in Facioscapulohumeral Dystrophy,” European Journal of Applied Physiology 125, no. 1 (2025): 157–165, 10.1007/s00421-024-05581-5. PMC11753324 39168898

[14] C. Bettio , V. Salsi , M. Orsini , et al., “The Italian National Registry for FSHD: An Enhanced Data Integration and an Analytics Framework Towards Smart Health Care and Precision Medicine for a Rare Disease,” Orphanet Journal of Rare Diseases 16, no. 1 (2021): 470, 10.1186/s13023-021-02100-z. PMC8567605 34736505

[15] G. Ricci , L. Ruggiero , L. Vercelli , et al., “A Novel Clinical Tool to Classify Facioscapulohumeral Muscular Dystrophy Phenotypes,” Journal of Neurology 263, no. 6 (2016): 1204–1214, 10.1007/s00415-016-8123-2. PMC4893383 27126453

[16] O. Crisafulli , R. Baptista , G. Bottoni , et al., “Unraveling the Link Between Resting Metabolic Rate and Phase Angle in Facioscapulohumeral Dystrophy: A Comparative and Associative Analysis,” European Journal of Applied Physiology 125, no. 12 (2025): 3507–3520, 10.1007/s00421-025-05853-8. 40522514

[17] O. Crisafulli , S. Sozzi , V. Quintiero , et al., “Interplay Between Balance, Gait Kinematic and Physical Activity Level in Facioscapulohumeral Muscular Dystrophy,” Scientific Reports 15, no. 1 (2025): 42973, 10.1038/s41598-025-26924-y. PMC12673088 41331267

[18] M. C. Barbosa‐Silva , A. J. Barros , J. Wang , S. B. Heymsfield , and R. N. Pierson, Jr. , “Bioelectrical Impedance Analysis: Population Reference Values for Phase Angle by Age and Sex,” American Journal of Clinical Nutrition 82, no. 1 (2005): 49–52, 10.1093/ajcn.82.1.49. 16002799

[19] J. Concato , P. Peduzzi , T. R. Holford , and A. R. Feinstein , “Importance of Events Per Independent Variable in Proportional Hazards Analysis. I. Background, Goals, and General Strategy,” Journal of Clinical Epidemiology 48, no. 12 (1995): 1495–1501, 10.1016/0895-4356(95)00510-2. 8543963

[20] R. O'Brien , “A Caution Regarding Rules of Thumb for Variance Inflation Factors,” Quality & Quantity 41 (2007): 673–690, 10.1007/s11135-006-9018-6.

[21] R. Mattiello , M. A. Amaral , E. Mundstock , and P. K. Ziegelmann , “Reference Values for the Phase Angle of the Electrical Bioimpedance: Systematic Review and Meta‐Analysis Involving More Than 250,000 Subjects,” Clinical Nutrition 39, no. 5 (2020): 1411–1417, 10.1016/j.clnu.2019.07.004. 31400996

[22] O. Di Vincenzo , M. Marra , A. M. Sacco , F. Pasanisi , and L. Scalfi , “Bioelectrical Impedance (BIA)‐Derived Phase Angle in Adults With Obesity: A Systematic Review,” Clinical Nutrition 40, no. 9 (2021): 5238–5248, 10.1016/j.clnu.2021.07.035. 34474193

[23] E. Mundstock , M. A. Amaral , R. R. Baptista , et al., “Association Between Phase Angle From Bioelectrical Impedance Analysis and Level of Physical Activity: Systematic Review and Meta‐Analysis,” Clinical Nutrition 38, no. 4 (2019): 1504–1510, 10.1016/j.clnu.2018.08.031. 30224304

[24] J. M. Statland , C. Heatwole , K. Eichinger , N. Dilek , W. B. Martens , and R. Tawil , “Electrical Impedance Myography in Facioscapulohumeral Muscular Dystrophy,” Muscle & Nerve 54, no. 4 (2016): 696–701, 10.1002/mus.25065. PMC4972708 26840230

[25] L. H. Wang , B. Sonbas Cobb , L. Riem , et al., “Electrical Impedance Myography Captures Features of Muscle Structure Measured by MRI and Transcriptomic Analysis in Facioscapulohumeral Muscular Dystrophy,” Journal of Neuromuscular Diseases 13, no. 3 (2026): 264–273, 10.1177/22143602251369246. PMC13438509 40920226

[26] K. Mul , C. Heatwole , K. Eichinger , et al., “Electrical Impedance Myography in Facioscapulohumeral Muscular Dystrophy: A 1‐Year Follow‐Up Study,” Muscle & Nerve 58, no. 2 (2018): 213–218, 10.1002/mus.26127. PMC6105423 29543984

[27] K. Mul , M. P. McDermott , R. J. Butterfield , et al., “Electrical Impedance Myography Detects Disease Progression Over 12 to 24 Months in Facioscapulohumeral Muscular Dystrophy,” Annals of Neurology 99, no. 6 (2026): 1545–1554, 10.1002/ana.78182. PMC13206185 41724900

[28] M. Yamada , Y. Kimura , D. Ishiyama , et al., “Phase Angle Is a Useful Indicator for Muscle Function in Older Adults,” Journal of Nutrition, Health & Aging 23, no. 3 (2019): 251–255, 10.1007/s12603-018-1151-0. PMC12280501 30820513

[29] T. Abe , Y. Yoshimura , R. Imai , Y. Yoneoka , A. Tsubaki , and Y. Sato , “Impact of Phase Angle on Physical Function in Patients With Acute Stroke,” Journal of Stroke and Cerebrovascular Diseases 30, no. 9 (2021): 105941, 10.1016/j.jstrokecerebrovasdis.2021.105941. 34217068

[30] X. Li , K. Zhang , Q. Guo , et al., “Impedance‐Derived Phase Angle as an Indicator of Physical Function in Maintenance Hemodialysis Patients,” Nutrition 136 (2025): 112795, 10.1016/j.nut.2025.112795. 40373354

