# Supplementary material for: Whole Body Phase Angle as a Promising Marker of Disease Severity in Facioscapulohumeral Muscular Dystrophy
Source: Muscle Nerve. 2026 Aug 31;74(5):1443–9. doi: 10.1002/mus.70391 (PMC13644891; doi:10.1002/mus.70391)
Supplement: Supplementary file 1 [file MUS-74-1443-s001.docx]

|  | Subgroup with physical activity level assessment | | | | |
| --- | --- | --- | --- | --- | --- |
|  | Total of subjects (N=38) | Male (N=22) | Female (N=16) | Category A (N=31) | Non-A category (N=7; all B) |
| *Sex* |  |  |  |  |  |
| Female | 16 (42%) | - | 16 (100%) | 15 (48%) | 1 (14%) |
| Male | 22 (58%) | 22 (100%) | - | 16 (52%) | 6 (86%) |
| *Age (years)* |  |  |  |  |  |
| Mean (sd) | 41.39 (14.98) | 38.81 (14.61) | 44.93 (15.23) | 41.06 (15.67) | 42.85 (12.41) |
| Anthropometric characteristics | |  |  |  |  |
| *Weight (kg)* |  |  |  |  |  |
| Mean (sd) | 71.22 (13.27) | 78.76 (10.58) | 60.86 (8.92) | 69.96 (13.01) | 76.81 (13.96) |
| *Height (m)* |  |  |  |  |  |
| Mean (sd) | 1.72 (0.09) | 1.77 (0.07) | 1.64 (0.06) | 1.71 (0.08) | 1.76 (0.15) |
| *Body mass index (kg/m^2^)* |  |  |  |  |  |
| Mean (sd) | 23.88 (3.13) | 24.93 (2.77) | 22.43 (3.11) | 23.73 (3.41) | 24.54 (1.36) |
| Phase angle (°) |  |  |  |  |  |
| Mean (sd) | 5.70 (1.14) | 6.15 (1.08) | 5.07 (0.92) | 5.53 (1.10) | 6.44 (1.08) |
| Clinical score (pts) |  |  |  |  |  |
| Mean (sd) | 4.50 (2.34) | 3.86 (2.23) | 5.37 (2.27) | 5.12 (2.09) | 1.71 (0.95) |
| Physical activity (METs-min/week) | |  |  |  |  |
| Mean (sd) | 2380.84 (1840.06) | 2789.70 (2128.63) | 1818.65 (1194.12) | 2305.95 (1980.75) | 2712.50 (1051.20) |

Supplemental Table 1. Descriptive statistics of the PAL-assessed cohort as whole group and stratified by sex and clinical phenotype.

Data are expressed as frequencies and mean (standard deviation). sd, standard deviation; kg, kilograms; m, meters; BMI, body mass index; pts, points; °, degree; METs-min/week, metabolic equivalent of task minutes per week; B, category B of the FSHD Comprehensive Clinical Evaluation Form.
